# The effects of delayed auditory feedback revealed by bone conduction microphone in adult zebra finches

**DOI:** 10.1038/srep08800

**Published:** 2015-03-05

**Authors:** Makoto Fukushima, Daniel Margoliash

**Affiliations:** 1 Department of Psychology, University of Chicago, Chicago IL 60637 U.S.A; 2 Department of Organismal Biology and Anatomy, University of Chicago, Chicago IL 60637 U.S.A

## Abstract

Vocal control and learning are critically dependent on auditory feedback in songbirds and humans. Continuous delayed auditory feedback (cDAF) robustly disrupts speech fluency in normal humans and has ameliorative effects in some stutterers; however, evaluations of the effects of cDAF on songbirds are rare. We exposed singing young (141–151 days old) adult zebra finch males to high-amplitude cDAF. cDAF exposure was achieved by the recording of bone-conducted sounds using a piezoelectric accelerometer, which resulted in high-quality song recordings that were relatively uncontaminated by airborne sounds. Under this condition of cDAF, birds rapidly (2–6 days) changed their song syllable timing. The one bird for which we were able to maintain the accelerometer recordings over a long period of time recovered slowly over more than a month after cDAF was discontinued. These results demonstrate that cDAF can cause substantial changes in the motor program for syllable timing generation over short intervals of time in adult zebra finches.

Songbirds are vocal learners and have been used as animal models for speech acquisition and production^1^,^2^. Auditory feedback (AF) is required for normal speech development and maintenance in humans^3^,^4^,^5^. In songbirds, AF is necessary for song development^6^,^7^,^8^ and adult song maintenance as demonstrated by experiments with deafened birds^9^,^10^. More recently, various real-time manipulations of AF have revealed the capability of the monitoring mechanism to adjust song morphology and sequences in the presence of altered sensory consequences of motor commands^11^,^12^,^13^.

Delayed auditory feedback (DAF) is known to be a robust method for inducing speech dysfluencies in humans, including a slowing of the rate of speech and stuttering^14^,^15^. However, attempts to investigate the effects of DAF on songbirds have been limited. Adult zebra finches can alter their syllable sequence after several weeks or more of exposure to syllable-triggered partial DAF^11^. Complete and continuous DAF (cDAF) playback to songbirds has also been previously attempted^16^, although the system used in those experiments could not produce high-amplitude DAF due to positive feedback constraints (reverberation). Thus, the effect of DAF on songbirds has yet to be evaluated with continuous, high-amplitude DAF that is similar to the cDAF implemented in studies that induce speech dysfluency in humans.

To circumvent these limitations of the previous studies, we developed a novel approach to implementing DAF that employed a small piezoelectric accelerometer attached to the skull. The accelerometer was relatively insensitive to airborne sound, which permitted making uncontaminated recordings of birds singing under high-amplitude altered feedback. The accelerometer recordings were used to provide feedback signals, delivered to the bird through a fixed speaker. In addition, we implemented frequency-shifted AF (FAF) with minimum time delay with the accelerometer recording, as a control with near-zero ms delay, and to compare its effect with adaptive morphological syllable changes previously reported in studies using a small headphone system^13^,^17^.

## Results

### DAF derived from bone conduction sounds in singing zebra finches

**Figure 1:**
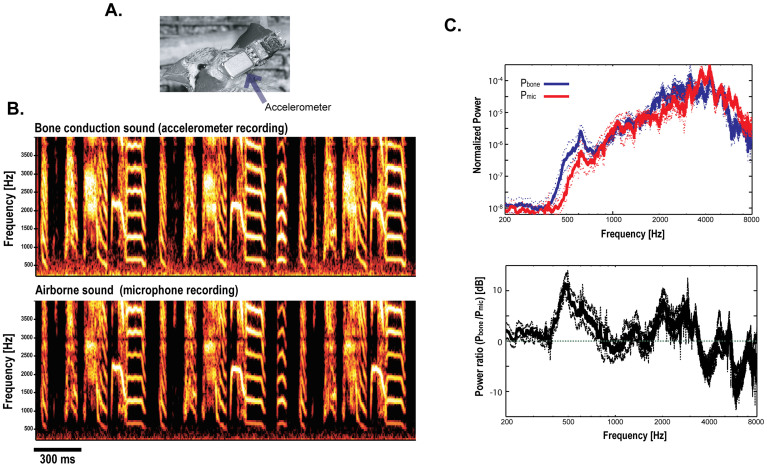
Examples of bone conduction sounds recorded using piezoelectric accelerometers that were chronically mounted to the skulls of zebra finches. (A) Picture of the accelerometer attached to the lateral surface of a zebra finch's skull. (B) Example spectrograms of a bone-conducted song of bird zf_yl11 recorded with an accelerometer (upper panel) and the same airborne song recoded by a microphone (lower panel). The recordings are shown for the frequency range below 4 kHz to facilitate evaluation of the similarity of the two spectrograms. The bone conduction song recording shows relatively higher power in the low frequency range (below 1 kHz) compared to the airborne recording. (C) Upper panel: The average of the power spectra of the bone-conducted and airborne songs recorded from five birds (solid line: average, dotted lines: ±SEM). For each bird, the spectrum was computed for syllables from 10 motifs and normalized by the power of the background noise level computed from silent periods (i.e., the log power noise was subtracted). The means and standard errors were computed from the log power spectra. The power spectrum of the airborne songs (P_mic_; red line) shows lower power over a range of low frequencies (circa 400 Hz–850 Hz) compared to the power spectrum of the bone conduction songs (P_bone_; blue line). Lower panel: The ratio between P_bone_ and P_mic_ was plotted in dB scale (i.e. 10 log_10_(P_bone_/P_mic_)). This comparison is valid between 200 and 4000 Hz (see Methods).

We recorded bone-conducted sounds from singing zebra finches using a piezoelectric accelerometer affixed to the lower layer of the skull (Figure 1A, see Methods). The bone-conducted signal provided high signal-to-noise recordings of songs over the broad frequency range of zebra finch songs (0.4–8 kHz; Figure 1B). Typically there was somewhat elevated noise at frequencies below circa 500 Hz recorded by the accelerometer than recorded by the microphone (Figure 1C; compare spectrograms in Figure 1B). Conversely, the signals recorded by the accelerometer did not suffer from minima caused by resonances in small sound booths that are commonly observed in microphone recordings. For example, note that the relative minima at 3 kHz throughout the microphone recording (Figure 1B, lower panel) is absent from the accelerometer recording (Figure 1B, upper panel).

The bone-conducted sounds had higher power in the low frequency range (<1 kHz) of the songs than the airborne sounds recorded by a microphone (Figure 1C, upper panel) even after taking into account the difference in the background noise level between the accelerometer and microphone recordings. This effect was most pronounced between 400 Hz and 800 Hz, a broad peak of increased power in the accelerometer recordings reaching a peak difference of about 10 dB at 500 Hz (Figure 1C, lower panel).

The power in the bone-conducted and airborne sounds was about the same above 800 Hz (Figure 1C, lower panel, see also Methods). In favorable preparations, high fidelity recordings from the accelerometer were maintained for one or more weeks. The degradation in recording quality resulted often from damage to the recording cable and detachment of the accelerometer from the bone caused by tissue regrowth.

**Figure 2:**
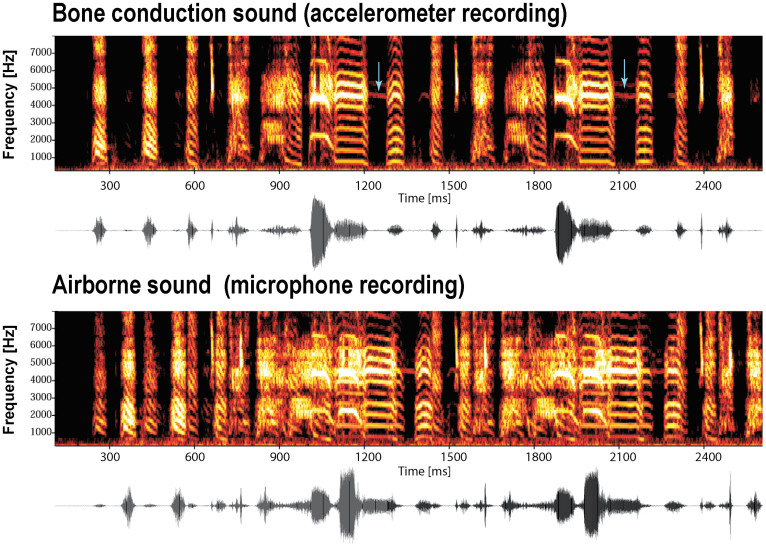
Bone-conducted and airborne sound recorded simultaneously during loud continuous DAF. The accelerometer recording of the bone-conducted sound during singing under loud continuous DAF (time delay of 100 ms) is shown in the top panel. The microphone recording of airborne sounds is shown in the bottom panel. The superposition of the bird's vocalization and the DAF from the speaker is noticeable. The accelerometer detected little of the DAF; for example, there are only a few low-amplitude delayed versions of the strongest signals visible in the spectrograms (which occur for the strongest spectral lines between 4 and 5 kHz; see the blue arrows). In contrast, the microphone recording clearly reflects two overlapping signals.

We took advantage of the fact that the accelerometer is relatively insensitive to airborne sound to produce high-amplitude cDAF while avoiding the positive feedback problem of traditional loud speaker-microphone systems^16^. This configuration also allowed us to obtain clean song recordings even during loud sound playback from the speaker. While the accelerometer recorded virtually uncontaminated versions of the bird's vocalization via bone conduction under DAF (Figure 2, top), the microphone recorded the superimposition of the bird's airborne vocalization and the DAF (i.e., the same song delayed by 100 ms) (Figure 2, bottom). Here, the sound intensity of the DAF played from the loud speaker was ~90 dB SPL at the microphone, which represented a 5.5-fold (~15 dB) increase in amplitude compared to the bird's vocalization at the microphone. We used this accelerometer recording to detect and analyze syllable sequences and morphologies during the distorted AF in the following experiments. The bird presumably experienced a mixture of normal feedback during singing (both airborne and including stimulation of the middle ear through substrate-borne vibrations), and airborne delayed feedback delivered by the speaker.

### Rapid syllable timing de-crystallization under loud DAF

**Figure 3:**
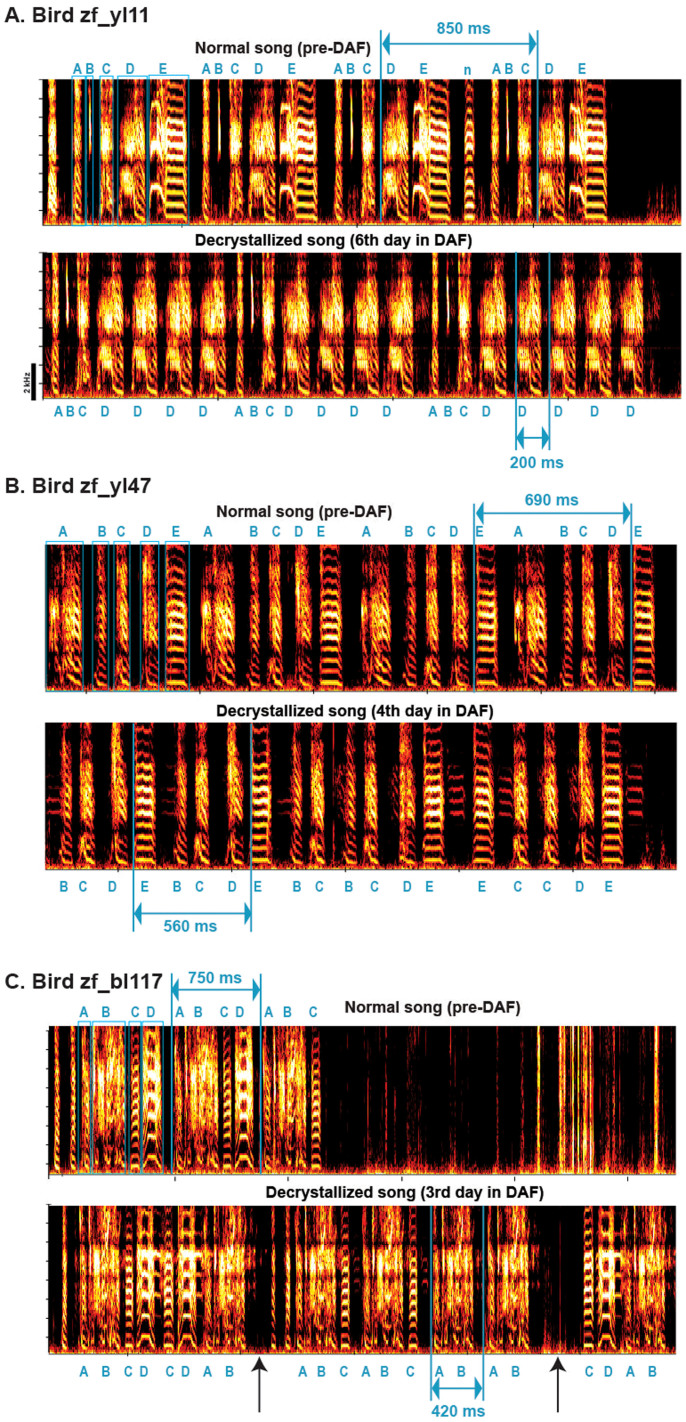
Exemplar song spectrograms before and after song de-crystallization from three birds exposed to DAF. In each pair of spectrograms ((A) zf_yl11, (B) zf_yl47, (C), zf_bl117), the upper trace represents a normal song prior to de-crystallization, and the lower trace represents a song with abnormal syllable sequence that was recorded after de-crystallization while the bird was experiencing DAF. All spectrograms were derived from the bone-conducted sounds recorded via accelerometers; see the text. The durations of the inter-onset intervals (IOIs) of the detected ("target") syllables are indicated by blue lines and text. These examples all show typical effects of the DAF treatment, including songs with normal as well as abnormal syllable sequences, and little apparent effect on syllable morphology even in the presence of large effects on syllable sequence. Note that the de-crystallized songs had missing syllables and, hence, shorter IOIs. Each syllable is labeled by an alphabet (A, B, C,…). “n” in the upper panel in (A) indicates a connecting note between motifs. All exemplars are of a single song bout except for the lower trace in panel (C), which has two inter-syllable intervals ≥200 ms (see arrows), hence three song bouts.

After a bird was implanted with an accelerometer, we waited several days until it fully recovered and began to sing readily. After recording a normal song baseline for 2–3 days, the birds were exposed to continuous DAF. Under these conditions, three adult birds (144–151 days old at the onset of DAF) exhibited abnormal changes in syllable timing by the second to sixth day after DAF onset (Figure 3). In preliminary experiments, an older adult bird and birds exposed to lower amplitude DAF showed less pronounced effects on their singing patterns, but we did not systematically evaluate the effects of age or amplitude of feedback.

The changes we observed included highly abnormal songs characterized by individual syllables repeated multiple times (similar to “stuttering”) (zf_yl11, Figure 3A), frequently dropping the first syllable (zf_yl47, Figure 3B), and dropping one or more syllables while repeating strings of other syllables (zf_bl117, Figure 3C). It should be noted that birds continued to sing normal songs and they interspersed normal and abnormal songs. While syllable sequences were substantially altered, the morphology of individual syllables remained relatively intact. For example, the identities of individual syllables before and after syllable timing changes can readily be visually matched (compare the upper and lower spectrograms of Figure 3A–C). We did not further quantify the morphological changes under DAF thoroughly, and some morphological changes might have occurred under DAF, as found in a previous study^11^.

**Figure 4:**
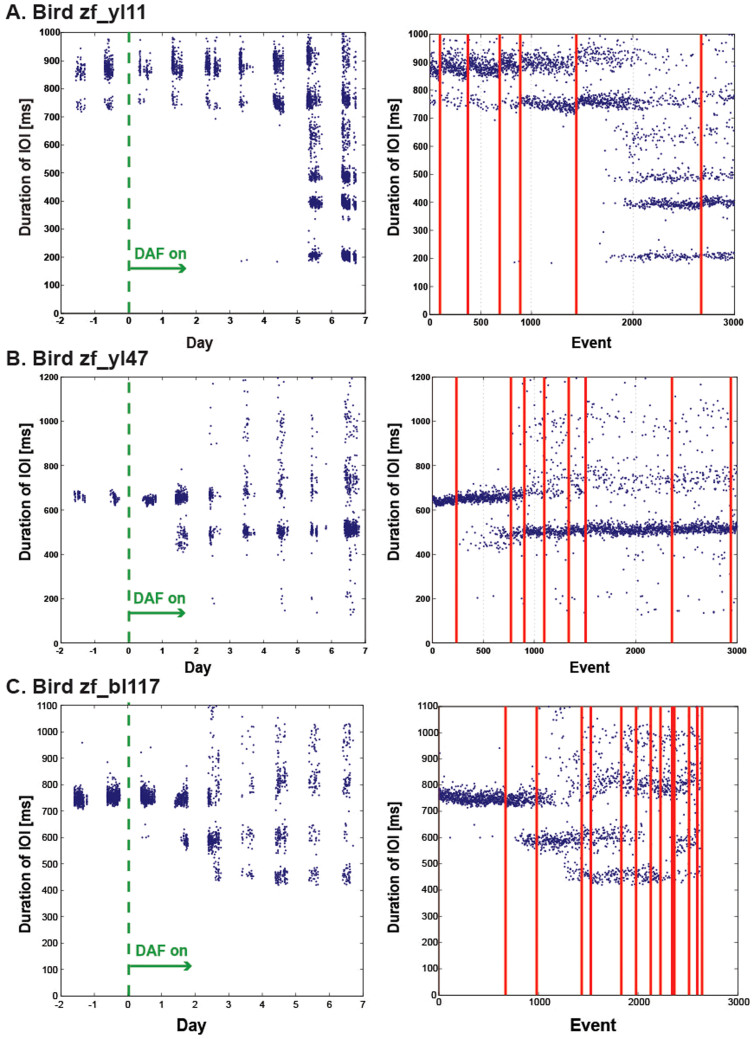
Changes in the distribution of the inter–onset intervals (IOIs) during the initial days of DAF exposure in three birds. The left panels show the durations of the IOIs plotted as a function of time (in days) for birds zf_yl11 (A), zf_yl47 (B), and zf_bl117 (C). Each dot corresponds to one IOI. Note there are one or two clusters of points in the IOI distributions prior to the onset of DAF. These clusters are maintained while additional ones appear two or more days after the onset of DAF. The right panels show data for the post-DAF period plotted as a function of the number of events (number of IOIs). The red lines correspond to boundaries between days. The data of the first 7 days of the right panels correspond to the data for Days 0–7 of the left panels.

To quantify the progression of song changes over time, we first chose a “target” syllable that could be most reliably detected automatically for each bird before and after DAF exposure (syllable “D” for zf_yl11, syllable “E” for zf_yl47, and syllable “A” for zf_bl117 in Figure 3). We then computed the time interval between the onsets of all target syllables (inter-onset-interval or IOI) detected within a given song bout. For normal adult zebra finches, the IOI distributions are usually clustered in one or two peaks, depending on the prevalence of a connecting note between motifs (see Methods). For birds with modified syllable timings, this approach can fail to detect cases when the target syllable is dropped, but has the advantage of not requiring faithful automated recognition of all syllables, which is technically challenging. Both manual inspections of songs produced under DAF and the large corpus of vocalizations that included the target syllables (Figure 4) indicated that the target syllables we chose were commonly sung under DAF.

The syllable timing change under DAF was reflected in changes in the IOI distributions. We observed increases and decreases in IOI. In principle, there can be many possible changes in song that could lead to such changes in IOI distributions. Increases in IOI could arise from occasional deletion of a target syllable or from insertion of additional syllables between target syllables. This can help explain the spread of longer IOIs and the long tails of the IOI distributions observed on the third day after DAF onset for birds zf_yl47 and zf_bl117 (Figure 4B and 4C).

We also observed, however, novel distinct peaks in the IOI distributions at shorter intervals that appeared for all three birds after the onset of DAF (Figure 4). Compared to the relatively minor changes at longer IOIs, these changes at shorter IOIs dominated the changes in IOI distributions for all three birds. The shorter IOIs could arise from birds simply dropping one or more syllables at specific points in the sequence. Alternatively, in principle birds could vary the length of existing syllables and/or introduce new syllables with a shorter duration than the original syllables – but only in a coordinated fashion to produce distinct peaks in the IOIs. The former explanation is more parsimonious, and is supported by extensive manual inspection of spectrograms that we conducted for each bird when first optimizing the automated analysis procedure to detect target syllables. Thus, the short-IOI peaks resulted from birds either deleting a syllable other than the target syllable, or repeating the target syllable. Indeed, visual inspection of spectrograms indicated that each distinct IOI peak was associated with one change in syllable sequence. For example, for zf_yl11, the short IOIs (at about 200 ms) that became prevalent at 6 days after DAF onset (Figure 4A, left panel) correspond to repetition of syllable “D” (Figure 3A, bottom panel).

It should also be noted that all three birds continued to occasionally produce normal syllable timings under DAF (Figure 4A–C). Thus, the effects of DAF were not to inexorably impose a single new song pattern, but rather to introduce new transitions with distinct probabilities. In this sense, continuous DAF induced singing patterns in these adult zebra finches more akin to those of Bengalese finch^18^. For two of the three birds (zf_yl47, zf_bl117), a distinct peak corresponding to abnormal syllable timing was observed by the second day of DAF exposure (Figure 4B and 4C, left column). The third bird (zf_yl11) also showed substantial numbers of abnormal songs, but only by the sixth day of DAF exposure (Figure 4A, left column). The two birds with rapid changes in syllable timing exhibited these changes after approximately 800 detected syllables produced under DAF (Figure 4B and 4C). The third bird (zf_yl11), with a longer onset prior to changes in syllable timing, exhibited novel syllable sequence after approximately 2,000 detected syllables produced under DAF (Figure 4A). We have no explanation of the differences across the three birds.

### Slow recovery from rapid de-crystallization

One obvious abnormality observed in zf_yl11 was that this bird repeated a single syllable type (syllable “D” in Figure 3A) multiple times in a row (“stuttering”), which can also be thought of as a deletion of all intervening syllables. The other two birds also deleted some, but not all, of the syllables between the target syllables used to calculate the IOI, and the identities of the deleted and repeated syllables varied between and within a given song bout (Figure 3B, C).

**Figure 5:**
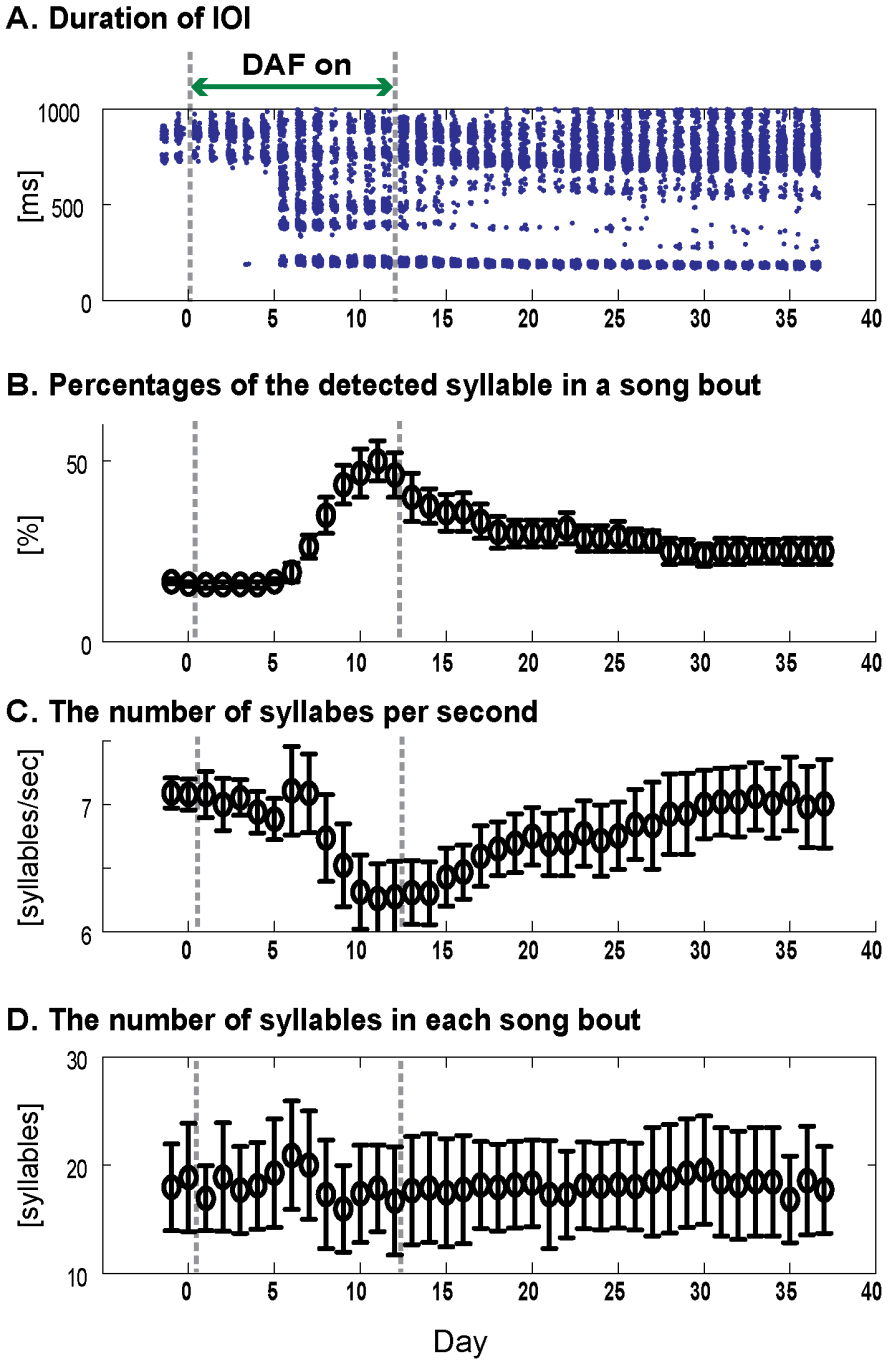
De-crystallization and recovery of syllable sequence for bird zf_yl11. The mean ±SD for each day is plotted. (A) Distribution of IOI durations. The data for the first nine days are identical to those in Figure 4. (B) Percentages of the detected ("target") syllable in a song bout as a function of the days. Note that the percentage starts to rise above baseline recordings on the sixth day of DAF, the same day abnormal syllables first appear in (A). (C) The tempo of a song bout. The tempo was defined as the number of syllables divided by the duration of a song bout (number of syllables/second). The change in tempo mirrors the change in the frequency of occurrence (B) of the target syllable. The reduction in tempo results from syllable D being of longer than average duration for the syllables of that song. (D) The number of syllables in each song bout.

Whereas “stuttering” in zf_yl11 emerged relatively abruptly, it was maintained stably over a long period of time. We were able to maintain stable accelerometer recordings from the bird (zf_yl11) for 39 days. The IOI distribution for this bird was then calculated over the entire period of song recording (Figure 5A). After the 2-day baseline song recording, this bird was exposed to DAF for 12 days. On the sixth day of DAF exposure, the syllable timing suddenly de-crystallized. This abnormal pattern was then maintained for more than one month, which included an extensive period of time after the cessation of DAF during which the bird experienced auditory feedback only from its own singing.

We extended our analysis on zf_yl11 to further characterize this song de-crystallization and recovery process in “stuttering”. First we counted the number of repeated syllables (syllable “D” in Figure 3A) in a song bout. A song bout was defined as a sequence of syllables with inter-syllable intervals <200 ms (see Methods). The percentage of the repeated syllable relative to all syllables increased from 16.6% in the baseline song to 50.1% by the 11^th^ day of DAF exposure (Figure 5B). This reflects the intense degree of stuttered song this bird produced, with song bouts dominated by sequential repetition of syllable “D” (Figure 3A, lower panel). After the cessation of DAF, this percentage slowly decreased over a month, indicating gradual recovery from the abnormal sequencing. Nevertheless, even 25 days after DAF had been turned off, on the last day we had recordings from this bird, still the recovery was incomplete. For example, the percentage of syllable “D” comprising all syllables of song bouts differed on the last day of recordings (24.3%) from that percentage in the two days of baseline recording prior to the onset of DAF (15.9%). The slow recovery of this bird is consistent with the slow recovery for zebra finch singing following long-term exposure to other forms of DAF^11^.

We also computed the “tempo” of a song as the total number of syllables in the song divided by the duration of the song bout, yielding a value of syllables per second. Figure 5C shows the mean tempos for each day for zf_yl11. The mean tempo of the baseline song was 7.1 syllables per second. The tempo decreased to 6.25 syllables per second by the 11^th^ day of DAF exposure. The change in tempo over days (increase followed by decrease) mirrored the percentage of syllables dominated by syllable “D” (compare Figure 5B and 5C). Thus, the song tempo decreased in “stuttering” presumably because the songs were more dominated by repetition of the syllable “D” whose duration is longer than those of the deleted syllables. The mean song tempo also gradually recovered to the baseline level over a period of more than a month, although recovery in this parameter was also incomplete, and the variance remained approximately five times greater than that of the singing recorded prior to the bird's initial exposure to DAF.

### Frequency-shift induced adaptive changes in the acoustic structure of song syllables

**Figure 6:**
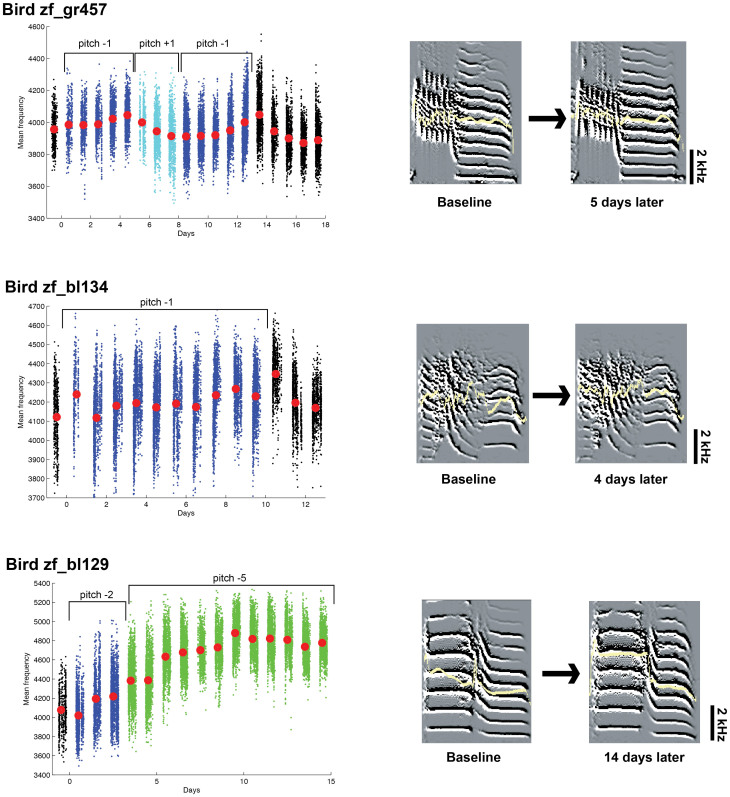
Change in mean frequencies of one syllable in each of three birds as induced by frequency–shifted auditory feedback. The three panels in the left column correspond to each of three birds. For each panel, each dot is calculated from a single rendition of the song syllable whose exemplar spectrogram is shown at right. Black dots indicate syllables that were sung with normal (unaltered) feedback. The red dots indicate the means of the mean frequencies from all syllables for each day. The right panel shows exemplar spectrograms of the song syllables prior to any FAF manipulations (baseline) and the indicated number of days after the start of FAF experiments. The mean frequencies are depicted by the yellow lines.

We also exposed an additional group of birds to continuous frequency-shifted AF (FAF), with FAF songs broadcast at the same loudness as for the DAF experiments. The system was able to introduce a frequency shift between what the bird sang and what was broadcast back to the bird. The system also introduced a small time delay (<10 ms). For two of the birds, the frequency of the AF was shifted downward (Figure 6, zf_bl134 and zf_bl129). Within a few days, the birds increased the mean frequency of their vocalizations; i.e., they altered their singing frequencies to compensate for the perceived reduction in frequency. To further characterize the dynamics of the response to FAF, we alternated the direction of the frequency shift of the AF provided to the third bird. Bird zf_gr457 was exposed to ±1 semitone-shifted AF for 13 days (−1 semitone shift for 5 days, +1 semitone for 3 days, and −1 semitone for 5 days). This bird was able to rapidly increase or decrease the mean frequency of its vocalizations to compensate for the changes in FAF. This was accomplished by changing the relative power at different frequencies (for example, different harmonics), not by changing the absolute frequencies of harmonics or other spectral features of song (Figure 6). Importantly, none of the three birds showed any evidence of changes in syllable timing in their songs that were induced by the same system when it was configured to present DAF. This demonstrates that short feedback delays, or other changes such as small spectral changes in feedback that the system may have introduced, did not induce syllable timing changes. Thus, the experimental variables under control – feedback delay for DAF experiments and frequency shift for FAF experiments – caused the observed changes in singing behaviors.

## Discussion

The current study took advantage of the insensitivity of accelerometers to airborne sounds to avoid the positive feedback problem of traditional loud speaker-microphone systems and realize high amplitude continuous DAF (cDAF). At unity or greater than unity gain, the coupling between the microphone and the loudspeaker would normally produce reverberation^16^. Our ability to record the birds' singing in the presence of high-amplitude song playback clearly demonstrated the advantages of using the accelerometer. In general, this method should be able to provide clean song recording under the presence of various types of noise or sounds (e.g. cage noise or songs from other birds^19^).

A pathway that couples vibrations of the larynx with the cochlea via the middle ear bones is well known in mammals; for example, in *Pteronotus parnellii*, the fundamental frequencies of echolocation calls are suppressed but stimulate auditory neurons^20^, and bone-conducted sounds in humans emphasize lower-frequency components compared to airborne sounds^21^. Here, we present the first report that robust bone-conducted sounds can be detected via an accelerometer attached to the skulls of singing birds. After 2–6 days of continuous exposure to the high-gain DAF achieved via the accelerometer recordings, adult zebra finches exhibited abnormal syllable sequencing. FAF exposure implemented using the accelerometer system induced compensatory morphological changes similar to those previously reported in studies using headphone systems in songbirds^13^,^17^.

Early studies in songbirds used deafening to alter auditory feedback^6^,^22^,^23^. Such studies on adult zebra finches found that their song morphologies and/or sequences changed slowly, about a month or more months after deafening^10^,^24^,^25^. The effect of deafening in adult zebra finches is dependent on the age of the bird, and younger birds tend to exhibit greater plasticity than older birds. The earliest changes in syllable sequence were observed in younger zebra finches (81–139 days old) and occurred as early as two weeks after deafening^25^. (Song crystallization and sexual maturity are thought to occur at about 90 days of age in zebra finches.) Deafening can induce clear changes in song morphology and sequence, although clear effects that are exclusive to syllable sequence have not been reported in deafening studies. A recent study reported changes in song *bout* sequence several weeks after deafening in young adult birds (92–138 days old), although changes in the sequences of the song motifs were limited to the birds that already repeated song syllables before deafening^26^. Another study reported changes in song sequencing in as little as 3 days post-deafening for two birds deafened at 103 days of age^24^. Those birds were younger than the birds exposed to cDAF in the current study (144–151 days old); nevertheless, our birds showed compelling changes in syllable sequence as early as the second day of cDAF.

DAF has been known to be a robust method for the induction of speech dysfluency for more than a half century^14^,^15^. In humans, headphone-microphone systems are normally used, in which one speaks into the microphone and then hears one's own voice in the headphones, with the delay to the headphone under experimental control. These systems commonly induce speech dysfluencies that include a remarkable slowing of the rate of speech and stuttering in subjects who attempt to maintain a normal rate of speech under DAF. Humans stutter at specific locations in speech, at the beginnings of phrases or sentences, and a substantial proportion of human stutterers enjoy significant if temporary amelioration from stuttering under conditions of DAF^27^. It would be interesting to test if DAF is able to induce a similar amelioration adult zebra finches whose song motifs ended with repeated syllables^28^. Notably the effects of lesion in the basal ganglia nuclei (Area X) in such adult birds induced significant song changes while Area X lesion do not cause song changes in normal adult birds^29^.

In previous experiments, singing adult zebra finches were exposed to delayed playback of subsets of song syllables^11^. In those experiments, two types of distorted AF protocols were used. In the first adaptive protocol, part of the bird's song was played back at a delay of 100 ms. In the second “syllable-triggered” protocol, only one target syllable was played back to the bird after a delay of 50 ms. After periods of 30–120 days in these environments, four out of five birds showed marked changes that included abnormal repetition (“stuttering”), addition, deletion, and distortion of song syllables. In the adaptive protocol, stuttering occurred in all three birds. Generally, these changes occurred within 42 days (6 weeks) of the onset of the protocol. In contrast, we demonstrated that birds exposed to cDAF altered their syllable sequences within as little as two days. This difference cannot be simply attributed to the effects of age because the ages of the birds we exposed to cDAF (144–151 days old at the onset of cDAF) overlap with the ages of the birds that were used in the aforementioned study (130–300 days old at onset)^11^. A second significant methodological difference between our study and previous DAF studies is that in previous studies, it was not possible to detect changes during the broadcasting of DAF. For example, in the previous study^11^, birds were permitted to sing without DAF for 10–15% of their song deliveries one day per week to evaluate song degradation. This approach creates tension between the desire to make longer recordings of singing without DAF to detect small changes in singing patterns and the desire to minimize exposure to normal feedback that might mitigate the effects of DAF. Even if the birds in the previous study^11^ altered their syllable sequences, it may have been difficult to detect syllable sequence changes using such a small proportion of the song samples. Indeed, our birds continued to produce normal syllable sequences even after they began to produce clearly abnormal syllable sequences after a short exposure to DAF. Alternatively, even the small number of songs delivered without abnormal DAF in the previous study^11^ may have helped to stabilize the normal song pattern, delaying the onset of DAF effects^30^. We have not systematically studied the effects of allowing birds to experience periods of normal feedback interposed with periods of continuous DAF, which could help define what are the critical features that induce the most rapid changes in behavior.

One previous study reported immediate effects of continuous DAF on the singing of adult zebra finches^16^. The predominant effect reported was difficulty in song initiation and truncation of the normal syllable sequence. We also observed this behavior in our birds at the onset of cDAF, although this behavior usually ceased after the birds habituated to cDAF and did not result in any significant change in IOI distributions. A similar behavior was also reported for Bengalese finches^31^. Thus, some but not all of the effects are shared between continuous DAF under conditions of lower amplitude^16^ and higher amplitude. Except for these minor changes, the birds in the current study did not show any changes in syllable sequence immediately after the onset of DAF exposure. In contrast, after the onset of sequence de-crystallization, the birds consistently produced abnormal syllable sequences, as indicated by the changes in IOI distributions (Figure 4). We note that de-crystallization occurred after at least one night's sleep in all cases. Sleep has been implicated in adult song maintenance^32^.

Observations of the single bird showing “stuttering” for which we were able to gather accelerometer recordings over an extended period of time revealed that recovery after the cessation of cDAF was slow, and was incomplete even after 1 month. This partial recovery took far longer than the 6 days of cDAF that were required to induce abnormal syllable sequence. This slow recovery is comparable to that found in the previous study using a form of continuous DAF^11^. These findings not only confirm that the song abnormalities we report here are different from transient or startle-like responses to unanticipated stimuli^16^,^33^ but also demonstrate that once abnormal singing behavior is induced by compensation to altered AF, recovery is very slow. Similarly, short exposure to altered visual feedback induced by displacement prisms can induce long-lasting effects on sensorimotor control in humans^34^,^35^.

The mechanism by which syllable sequences are changed by DAF is a matter of speculation. Even in a normal environment, there is always a time delay during which sound produced from the vocal organs travels to the song system. The song system must cope with this delay to achieve normal song development and song maintenance^36^,^37^,^38^,^39^. In zebra finches, HVC neurons integrate information over hundreds of milliseconds^40^, with each projection neuron bursting at one particular position in the motif and with interneurons tending to be suppressed when projection neurons burst^39^,^41^,^42^. HVC neurons are also highly selective for the bird's own song over virtually any conspecific song or artificial stimuli^39^,^43^,^44^,^45^ so that playback of own song via DAF may be a particularly potent stimulus. Given this, we speculate that, in our experiments, the DAF had its principled effect by altering the chains of activity of HVC neurons that are hypothesized to generate sequences of syllables^37^.

## Methods

All animal procedures were performed according to protocols approved by an Institutional Animal Care and Use committee at the University of Chicago and were consistent with National Institutes of Health guidelines.

### Animals

Adult zebra finches were obtained from our breeding colony at the University of Chicago. Three of the birds that were exposed to DAF were adults (141–151 days old) at the start of DAF. Three other birds were exposed to frequency-shifted AF (FAF). They were 124, 163, and 205 days old at the start of FAF. One additional bird was used to evaluate accelerometer recordings, independent of AF manipulations (Figure 1).

### Experimental procedure

Each bird was placed in a small sound-attenuation chamber (AC-1; Industrial Acoustics Corporation, NY, USA). Bone-conducted songs were recorded using piezoelectric accelerometers (BU-1771 or 7135, Knowles Acoustics, IL, USA) that were chronically affixed onto the skulls of the zebra finches (Figure 1A). The accelerometers were positioned close to the left ear canal. Prior to implanting the accelerometer, the birds were deprived of food and water for 1 hr and then anesthetized with a 50 ml intramuscular injection of Equithesin. After removing the top layer of the skull, the accelerometer with a connector was attached to the bottom layer of the skull with cyanoacrylate glue first, and it was then secured further with dental acrylic. After the birds recovered, the connector was attached to a cable that was connected to an amplifier via a slip ring commutator (Dragonfly Inc., VA, USA). The airborne songs of birds were recorded using an omni-directional microphone (AT803B, Audio Technica) and digitally saved to a computer disk (sampling rate, 20 kHz; band-pass filtering, 200 Hz–10 kHz). The accelerometers had frequency responses of approximately ±1 dB over the range of 50–4000 Hz. The microphones had frequency responses of approximately ±1 dB over the range of 150–4000 Hz, with an 8 dB peak at 9 kHz. Therefore, the comparison between the airborne and bone conduction sounds in Figure 2 is valid between 200–4000 Hz in which both frequency responses from the microphone and accelerometer are similarly flat.

To achieve continuous DAF, bone-conducted sound was continuously played back from a loud speaker with a time delay of 100 ms. The signal delay was achieved using a computerized system that digitally recorded sounds at 50 kHz with 16-bit resolution on a Linux operating system (DaqBoard/3000; IOtech Inc., OH, USA). For FAF, the bone-conducted sounds were processed with a frequency shifter (PS-5, Roland Corp. Japan) and played back via the loudspeaker. The frequency shifter induced a small delay (<10 ms). We used a range of frequency shifts from −5 to +1 semitone. For all playbacks (DAF and FAF), the RMS amplitude of the altered feedback at the microphone was 5–6 times higher than that of bird's vocalization at the microphone. The sound pressure level of the altered feedback was approximately 90 dB SPL during singing. This is comparable to the sound pressure level used for partial DAF in a previous study^11^. We cannot exclude the possibility that the birds could still hear normal auditory feedback under the high-gain DAF and thus the effect could be caused by the overlap between normal and delayed AF.

### Data analysis

Vocal recordings were processed using Sound Analysis Pro (SA+) software^46^ by segmenting the continuous sound recordings to compute several acoustic features associated with each segmented sound. The detection of song syllables was performed using either a clustering program in SA+ or a syllable identification method^31^ based on the computation of the Mahalanobis distances between segmented sound vectors and manually labeled syllable vectors using acoustic feature values computed in SA+.

To quantify changes in syllable sequencing in three birds exposed to DAF, we first identified the song syllable that was detected most reliably among all syllables by the clustering algorithm (1 out 4 syllables for zf_bl117, 1 out of 5 syllables for zf_yl11, and zf_yl47). Then, we computed the time intervals between the onsets of the syllable (inter-onset intervals or IOIs). Each IOI was calculated within a song bout that was defined as a sequence of syllables with intervals less than 200 ms. That is, each song bout was separated from other bouts by at least 200 ms. For example, using this definition of a bout, the top panels in Figure 3A, 3B, and 3C include one song bout for each of the three birds, whereas the lower panels include one song bout for zf_yl11 (Figure 3A), one for zf_yl47 (Figure 3B), and three for zf_bl117 (Figure 3C). Normally, adult zebra finches sing highly stereotypical songs in which a given syllable is produced once per motif (i.e., the sequence of syllables is repeated). These songs result in IOI distributions with single peaks or, occasionally, two peaks if the bird occasionally introduces a “connecting note” between motifs. Some, but not all, birds introduce this “connecting note” (Figure 3A).

To analyze changes in three birds exposed to FAF, we computed the mean frequencies of song syllables with SA+, which was an estimate of the central tendency of the derivative power distributions^46^. We used a syllable most reliably detected by SA+ for the analysis in Figure 6 (1 out 4 syllables for zf_bl134, 1 out of 5 syllables for zf_gr457, and 1 out of 6 syllables for zf_bl129).

## Author Contributions

M.F. performed research and analyzed data; M.F. and D.M. wrote the main manuscript text. M.F. prepared all figures. All authors reviewed the manuscript.
